# *Antrodia cinnamomea* Enhances Chemo-Sensitivity of 5-FU and Suppresses Colon Tumorigenesis and Cancer Stemness via Up-Regulation of Tumor Suppressor miR-142-3p

**DOI:** 10.3390/biom9080306

**Published:** 2019-07-25

**Authors:** Yan-Jiun Huang, Vijesh Kumar Yadav, Prateeti Srivastava, Alexander TH Wu, Thanh-Tuan Huynh, Po-Li Wei, Chi-Ying F. Huang, Tse-Hung Huang

**Affiliations:** 1 Department of Surgery, College of Medicine, Taipei Medical University, Taipei 110, Taiwan; 2 Division of Colorectal Surgery, Department of Surgery, Taipei Medical University Hospital, Taipei Medical University, Taipei 110, Taiwan; 3 The Division of Translational Medicine, Graduate Institute of Biomedical Informatics, Taipei Medical University, Taipei 110, Taiwan; 4 The PhD Program for Translational Medicine, College of Science and Technology, Taipei Medical University and Academia Sinica, Taipei 110, Taiwan; 5 Graduate Institute of Medical Sciences, National Defense Medical Center, Taipei 114, Taiwan; 6 Center for Molecular Biomedicine, University of Medicine and Pharmacy, Ho Chi Minh City 217, Vietnam; 7 Institute of Biopharmaceutical Sciences, National Yang Ming University, Taipei 112, Taiwan; 8 Department of Traditional Chinese Medicine, Chang Gung Memorial Hospital, Keelung 204, Taiwan; 9 School of Traditional Chinese Medicine, Chang Gung University, Taoyuan 204, Taiwan; 10 School of Nursing, National Taipei University of Nursing and Health Sciences, Taipei 23741, Taiwan; 11 Graduate Institute of Health Industry Technology, Chang Gung University of Science and Technology, Kweishan, Taoyuan 333, Taiwan

**Keywords:** colon cancer, *AC*, 5-FU, EMT, stemness, miR-142-3p

## Abstract

5-Fluorouracil (5-FU) regimen remains the backbone of the first-line agent to treat colon cancer, but often these patients develop resistance. Cancer stem cells (CSC’s) are considered as one of the key contributors in the development of drug resistance and tumor recurrence. We aimed to provide preclinical evidence for *Antrodia cinnamomea* (*AC*), as a potential in suppressing colon cancer CSC’s to overcome 5-FU drug-resistant. In-vitro assays including cell viability, colony formation, *AC* + 5-FU drug combination index and tumor sphere generation were applied to determine the inhibitory effect of *AC*. Mouse xenograft models also incorporated to evaluate in vivo effect of *AC*. *AC* treatment significantly inhibited the proliferation, colony formation and tumor sphere generation. *AC* also inhibited the expression of oncogenic markers (NF-κB, and C-myc), EMT/metastasis markers (vimentin and MMP3) and stemness associated markers (β-catenin, SOX-2 and Nanog). Sequential treatment of *AC* and 5-FU synergized and reduces colon cancer viability both in vivo and in vitro. Mechanistically, *AC* mediated anti-tumor effect was associated with an increased level of tumor suppressor microRNAs especially, miR142-3p. *AC* can be a potent synergistic adjuvant, down-regulates cancer stemness genes and enhances the antitumor ability of 5-FU by stimulating apoptosis-associated genes, suppressing inflammation and metastasis genes through miR142-3p in colon cancer.

## 1. Introduction

The prevalence of colorectal cancer (CRC) is expected to be higher than almost 2.2 million, it is expected that by 2030, the new cases and mortality would be around 1.1 million globally [1]. Surgical resection remains the best option for treating the patients with stage II~III colon cancer, but 30–50% of these patients have tumor relapse even after resection and is found to be related with the higher risk of cancer-associated death [2,3]. Most of these recrudescences arise during the period of the first 2 years after the surgery [4]. Despite taking effective measures of cancer surveillance, and major advancement in treatment modalities including chemotherapy and target therapy. The drug resistance in colon cancer still remains a leading hindrance to successful chemotherapy. Recent studies have been associated with Cancer stem cells (CSC’s), which are a small group of cancer cells having the potential to undergo for self-renewal, differentiation and tumor initiation abilities [5]. CSC’s are also thought to be the key player involved in the failure of cancer therapy due to their substantial chemo-resistance properties that eventually leads to disease recurrence and metastasis [6]. More reports have surfaced to indicate for improving the cancer patient’s outcomes, traditional therapies approaches should be now shifted to targeting the specific CSC’s [2]. Therefore, therapeutic approaches that can also target CSC’s are being pursued to decreases the risk of cancer recurrence and metastasis.

It has been seen the effect of compounds isolated from natural source gives better response to cancer therapy, such as Stictic acid a secondary metabolite from Lichen *Lobaria pilmonaria* inhibits the growth of malignant cells and shows the anticancer effect in human colon adenocarcinoma [7]. Phytol is a diterpene alcohol from chlorophyll induces the concentration-dependent cell cytotoxicity in human cancer cell lines [8]. 

*Antrodia cinnamomea* (*AC*) is a Taiwan endemic species belonging to the genus *Antrodia* (Polyporaceae). *AC* is a photophobic parasitic fungus developing in the inner heartwood wall of *Cinnamomum kanehirai* Hayat (Lauraceae) [9]. It has been used by Taiwanese as a traditional medicine for the treatment of several different ailments, such as diarrhoea, liver disease, hypertension, inflammation, etc. for long times in the history [10]. Isolated compounds from *AC* are reported to induce apoptosis on colon cancer cells (HT-29 and SW-480) [11]. Other studies also revealed that *AC* induces apoptosis in human liver and oral cancer cells via mitochondrial-dependent pathways [12,13]. Recent studies also indicate that *AC* not only causing the apoptosis to tumor cell, but also protects liver cells from free radical-induced oxidative stress through the Nrf-2 activation and up-regulation of the MAP kinase-mediated antioxidant genes [14,15]. Furthermore, *AC* inhibits the proliferation of head and neck cancer cells and migration of leukaemia cells [16,17,18,19]. These findings strongly suggest *AC* can be a potential source of new cancer therapeutics. In our research, we aimed to provide experimental supports for inhibiting colon tumorigenesis using *AC*. We demonstrated *AC* treatment was able to reduce the colon tumorigenesis, not only in vitro but also in vivo. Notably, *AC* treatment suppressed CSC’s properties via the downregulating oncogenic (NF-kB, c-Myc), EMT (vimentin) and stemness (β-catenin, Sox2, Nanog) associated markers. 

## 2. Materials and Methods

### 2.1. Cell Culture, Chemicals and Reagents

The human colon cancer cell lines (SW480, SW620 and HCT116) were purchased from the American Type Culture Collection (ATCC), and cells were cultured according to ATCC’s recommend culture conditions. Colon sphere formation assay was performed according to a previously described method [20]. SW480, SW620 and HCT116 were cultured in Serum-Free Medium composed of DMEM/Ham’s F12 (1:1), human epidermal growth factors (hEGF, 20ng/mL), basic fibroblast growth factor (bFGF; 10ng/mL (PeproTech, Rocky Hill, NJ, USA), 2 ug/mL of 0.2% heparin (Sigma), and 1% penicillin/streptomycin (P/S, 100 U/mL, Hyclone). Cells were seeded (1000 cells/mL) in 12-well low adhesion plates and incubated at 37 °C and 5% CO_2_ for 5–7 days. Cells aggregates or spheroids which are compact, spherical, non-adherent massed greater than 50 µM in diameter were counted. 5-FU was purchased from SelleckChem, Taiwan (Cat. No. S1209). The mycelia of *AC* were kindly provided by Balay Biotechnology Corporation (Taipei, Taiwan, Republic of China). The *AC* extract was isolated according to previously established methods with some modifications [21]. The mycelia of *AC* were air-dried and extracted with boiling water (at the ration of 1:20, *w*/*v*) for 6 h. The liquid and precipitate were then separated by centrifugation and the suspension (non-soluble matters) was filtered out. The filtrate was then mixed with 4 volumes of ethanol (95%) overnight to precipitate crude extract. The crude extract was then spun at 4000× *g* for 30 min to remove the supernatant. This crude extract was termed aqueous *AC* mycelia extract and were dissolved in water and stored at room temperature for further analysis. The quality control was done by measuring the sugar content of the *AC* extract where the average concentration of polysaccharide is 4.00 ± 0.80 mg/g.

### 2.2. Cell Viability Test

Sulforhodamine B (SRB) assay [22] was used to determine cellular viability. Briefly, the colon cancer cells or colon spheres were seeded in 96 well plates (3.5 × 10^5^ cells/well) and treated with drugs (*AC* or 5-FU) alone or in combination at the specified concertation and times. After the respective drug treatments relative cell number was estimated by SRB reagent according to the manufacturer’s protocol (Sigma, Ronkonkoma, NY, USA). 

### 2.3. Apoptosis Assay

Annexin V-PI+ viable cells and Annexin-V+ apoptotic cells were estimated by flow cytometry and the later were considered apoptotic cells. Data were collected in FACS Calibur (Becton-Dickinson, Mountain View, CA) and analyzed by using the Cell Quest software (Becton-Dickinson), these experiments were performed thrice. 

### 2.4. RNA Isolation, RT-PCR and Quantitative RT-PCR

TRIzol-based protocol (Life Technologies) was used to isolate total RNA and purify according to manufacturer instructions. One microgram of total RNA was reverse transcribed using QIAGEN OneStep RT-PCR Kit (QIAGEN, Taiwan), and the PCR reaction was carried out using a Roto-Gene SYBR Green PCR Kit (400, QIAGEN, Taiwan). The primer sequence for genes, SOX2, forward: 5′-AAATGGGAGGGGTGCAAAAGAGGAG-3′ and reverse: 5′-CAGCTGTCATTTGCTGTGGGTGATG-3′. Nanog, forward: 5′-AATACCTCAGCCTCCAGCAGATG-3′ and reverse: 5′-TGCGTCACACCATTGCTATTCTTC-3′. β-catenin, forward: 5′-ACTGGCAGCAACAGTCTTACC-3′ and reverse: 5′-TTTGAAGGCAGTCTGTCGTAAT-3′. The primer sequences internal control RPLP0 were: forward: 5′-TGGTCATCCAGCAGGTGTTCGA-3′ and reverse: 5′-ACAGACACTGGCAACATTGCGG-3′. List of microRNA primer sequences was enumerated in Supplementary Table S1.

### 2.5. Colony Formation Assays 

The colony-forming assay was executed according to the previously explained protocols [23] with some modifications. Briefly, a total of 500 colon cancer cells were seeded in 6 well plates and treated with *AC* (40 μg/mL equivalent of IC20 values). The cells were allowed to grow for another week and then collected, fixed, and counted.

### 2.6. SDS-Page and Western Blotting 

Total protein lysate of colon cancer cells was extracted after the treatments from different experiments were separated using the SDS-PAGE using Mini-Protean III system (Bio-Rad, Taiwan) and transferred onto the PVDF membranes using Trans-Blot Turbo Transfer System (Bio-Rad, Taiwan). The membranes were incubated for overnight at 4 °C with respective primary antibodies PARP (#9542P), β-catenin (#9562), vimentin (#5741P), NF-κB (#6956S), β-actin (Sc-47778), C-myc (Sc-40), MMP3 (Sc-6839), ABCG2 (10051-1-AP). Secondary antibodies purchased from Santa Cruz Biotechnology (Santa Cruz, CA, USA) and ECL detection kit was used for the detection of the protein of interests. The image was captured and analyzed using UVP BioDoc-It system (Upland, CA, USA).

### 2.7. ALDEFLUOR Assay and ALDH1+ Population Cell Sorting by FACS 

The Aldehyde Dehydrogenase 1 (ALDH1) enzymatic activity showing positive cell populations were isolated according to the instruction of the manufacturer (STEMCELL Technologies, Durham, NC, USA). The human colon cancer cells (SW480, SW620 and HCT116) were suspended in the concentration of 1 × 10^6^ cells/mL in ALDEFLUOR assay buffer containing ALDH substrate (BAAA, 1 µmol/L per 1 × 10^6^ cells) and incubated for 40 min at 37 °C. Cells incubated with ALDEFLUOR substrate 50 mmol/L diethylaminobenzaldehyde (DEAB) as reference control, a specific ALDH inhibitor. TO eradicate contamination of cells of mouse origin from the xeno-transplanted tumors, we used staining with anti-H2Kd antibody (BD biosciences, 1/200, 20 min on ice) followed by staining with a secondary antibody labelled with phycoerythrin (PE) (Jackson labs, 1/250, 20 min on ice). The sorting gates were established using as negative controls the cells stained with PI only, for viability, the ALDEFLUOR stained cells treated with DEAB and the staining with secondary antibody alone.

### 2.8. In Vivo Studies 

All the animal experiments and maintenance conformed to the strict compliance to the animal use protocol from Taipei Medical University (protocol LAC-2014-0170). Female NOD/SCID mice were purchased from BioLASCO Taiwan Co., Ltd. Tumor-initiating ability test was first to assess by using the tumor spheres generated from the naïve DLD-1 cells. DLD-1 spheroids cells (1 × 10^6^ cells/injections) were subcutaneously injected into the right flanks of NOD/SCID mice. 

Second, for drug treatment test, subcutaneous tumor models were established using the tumor sphere grown from DLD-1 (1 × 10^6^ cells/20µL/injection) in NOD/SCID mice (4–6 weeks old). The treatment commenced when the tumor became palpable. For assessing the effect of *AC* on tumor growth and 5-FU toxicity. The mice were divided into different groups with matched weight, first the control group receiving the normal saline (vehicle group), second group treated with 10 mg/kg 5-FU, third group treated with 50mg/kg *AC*, and fourth group treated with 10 mg/kg 5-FU + 50 mg/kg *AC* (5-FU + *AC* group), 

Dosing regimens are as the following, 5-FU alone (10 mg/kg, two times/week), *AC* alone (50 mg/kg, five times/ week), and 5-FU + *AC* combination (10 mg/kg, two times/week; 50mg/kg five times/week, respectively). All the treatments were given intraperitoneal. The change in tumor burden was expressed in fold change in cubic centimeter as compared to its starting volume. 

Mice were sacrificed humanely upon the completion of the experiments, and tumor biopsies were collected for further analysis. 

### 2.9. Statistical Analysis

All experiments were executed in triplicates. Statistical analyses were performed using Student’s *t*-test by GraphPad Prism software, where a *p*-value < 0.05 was considered as statistically significant and was indicated with an asterisk.

## 3. Results

### 3.1. AC Inhibits the CRC Tumorigenesis and Colon Sphere Formation

**Figure 1:**
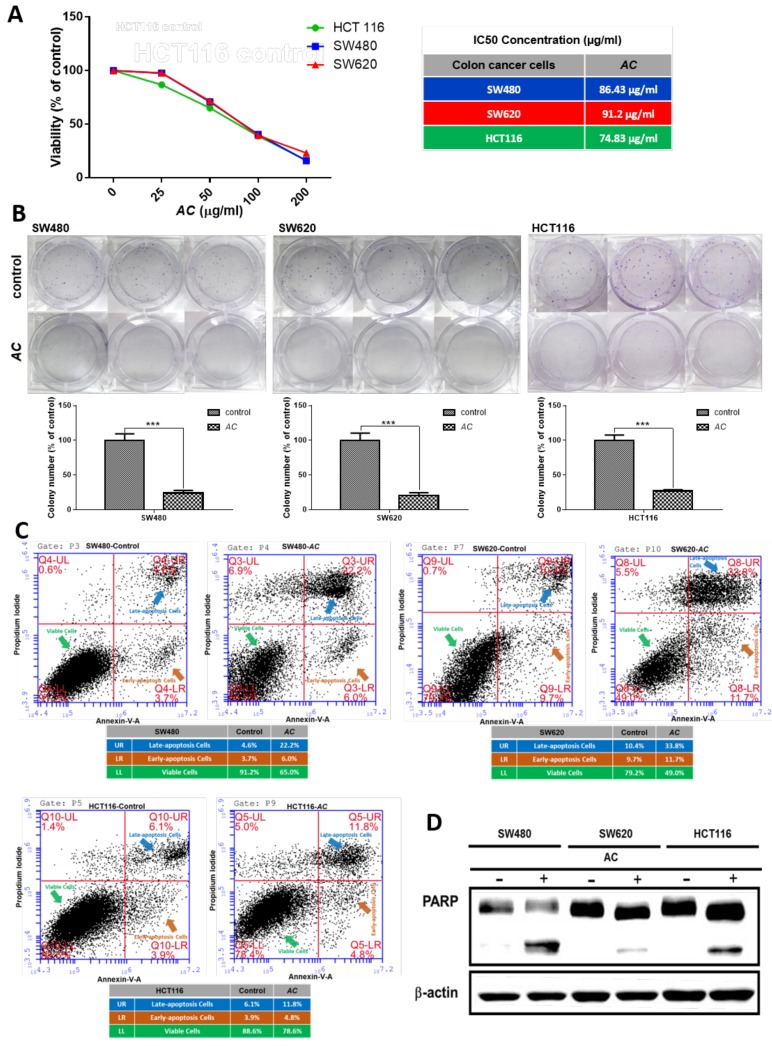
*AC* treatment decreases the viability of CRC cells by inducing apoptosis. (**A**) Cell viability assay demonstrates that *AC* is effectively suppressing the cellular viability in CRC cells. The IC50 values of *AC* in all three cells (SW480, SW620 and HCT116) are indicated. (**B**) *AC* effectively suppresses colony-forming ability. *** *p* < 0.001. (**C**) Colon cancer cell lines were treated with *AC* (100 µg/mL) for 24 h and then analyzed for apoptosis by flow cytometer for Annexin-V+ and PI+ stained cells. *AC* significantly induces apoptosis in CRC cells (SW480, SW620 and HCT116). Number in the box indicates the percentage of Annexin-V+ cells (control versus *AC* treatment, left and right respectively). (**D**) Western blots of the whole-cell lysates from *AC* treated colon cancer cells showed a significantly increased level of cleaved PARP (pro-apoptotic marker).

Mechanism of apoptosis was found connected with the advancement and progression of CRC’s [24]. *AC* treatment was reported to have the ability to suppress tumor growth in various cancers [16,25]. Anti-cancer effect of *AC* on colon cancer cells were determined by the cellular viability and colony formation assay by SRB test. *AC* treatment observed to reduces or inhibits the lung cancer cell viability by inducing apoptosis [26]. For further investigation of the effect of *AC* on CRC cells, apoptosis was examined through Annexin-V-FITC Assay [27]. We first observed *AC* effectively inhibits the cell viability of SW480, SW620 and HCT116 colon cancer cells as demonstrated in Figure 1A. Furthermore, *AC* treatment also significantly inhibited colony formation (Figure 1B). We subsequently found that *AC* treatment significantly induced apoptosis in CRC (SW480, SW620 and HCT116) cells. *AC* treated samples also exhibited higher percentage of Annexin-V positively stained cells (SW480: 4.6% vs 22.2%; SW620: 10.4% vs 32.8%; HCT116: 6.1% vs 11.8%, control versus *AC* treatment, respectively) (Figure 1C). The increased apoptosis was associated with increased expression of cleaved PARP (pro-apoptotic marker) (Figure 1D).

### 3.2. AC Suppresses the Tumorsphere Formation and Reduces the ALDH1+ and Side Population of Stem Likes Cells

**Figure 2:**
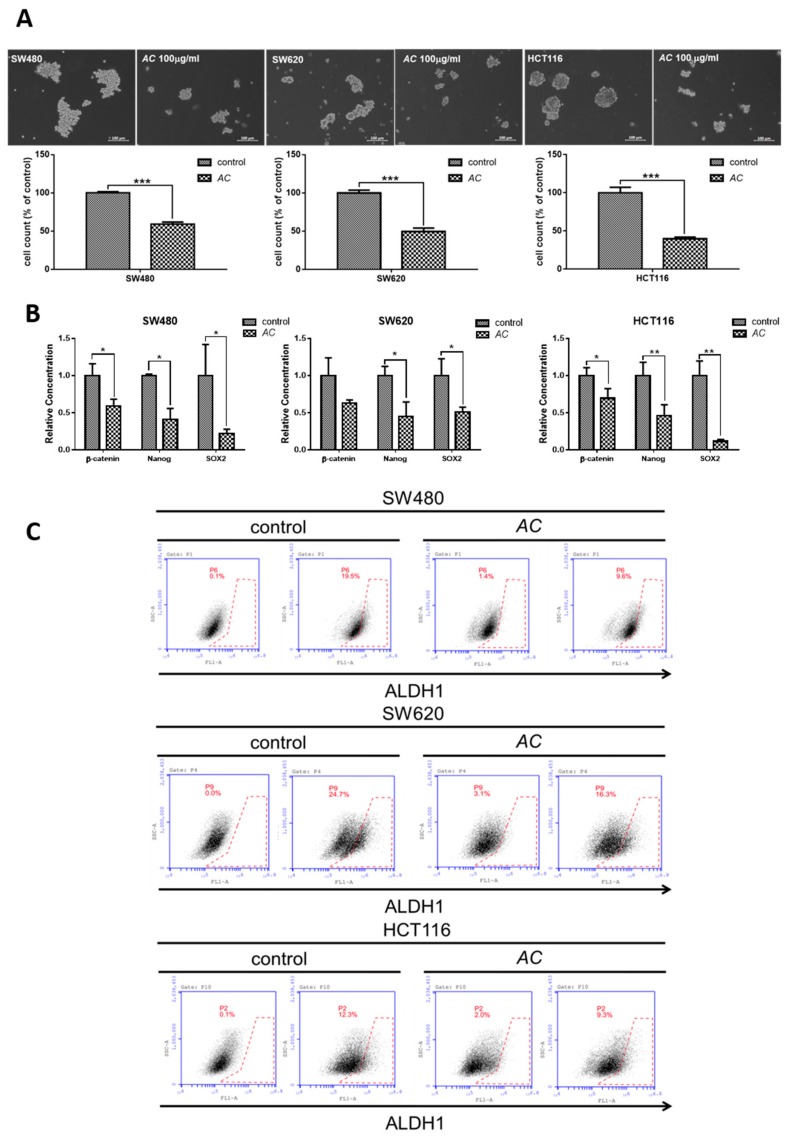
Suppression of cancer stemness by *AC* treatment. (**A**) Tumor sphere formation assay. Colon cancer cells treated with *AC* (100 µg/mL, 48 h) demonstrated a significant reduction in number of tumor spheres generated under serum-deprived culture conditions. *** *p* < 0.001. (**B**) q-PCR analysis showed that *AC* treatment down-regulated cancer stem cell markers β-catenin, SOX-2, Nanog. ** *p* < 0.01, * *p* < 0.05. (**C**) Aldefluor assay showed that *AC* treatment (100 µg/mL, 48 h) prominently and dose-dependently reduces the ALDH1 enzymatic activity in all three colon cancer cell lines examined.

CSC’s are a group of cells within a tumor mass having the sphere-forming and high self-renewal ability, resulting in chemo-resistant properties [28]. ALDH1+ cells expression associated with cancer recurrence and poor prognosis in human cancers stems cells [29]. We first identified that *AC* suppresses the CRC cells self-renewal and tumor initiation ability when grown under the serum-starved culture condition as exhibited by significantly reduced colon sphere-forming capacity (Figure 2A). Anti-cancer effect of *AC* acting on CSC’s was related with reduced expression of cancer stem cell markers such as β-catenin, SOX-2, and Nanog (stemness marker) in comparison to control (Figure 2B). We subsequently showed SW480, SW620 and HCT116 colon cancer cells treated with *AC* (100 µg/mL, 48 h) prominently and dose-dependently reduced the ALDH1 activity (Figure 2C).

### 3.3. AC Treatment Increased 5-FU Sensitivity In Vitro

**Figure 3:**
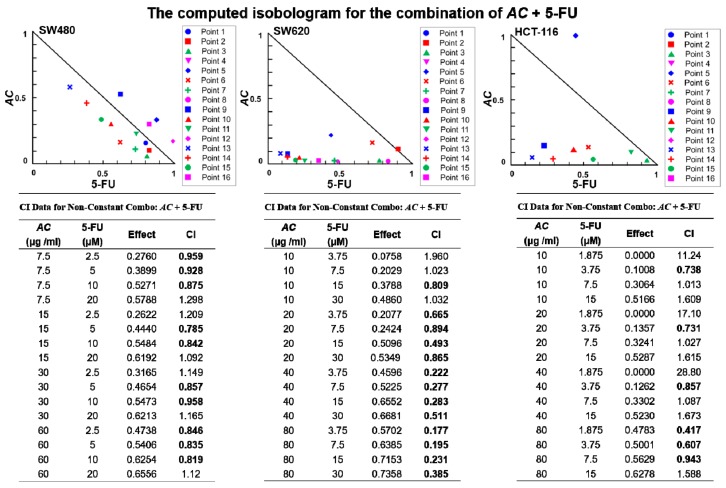
*AC* treatment increased 5-FU sensitivity in colon cancer cells. Drug combination assay, different concentrations of *AC* and 5-FU were used in combination for calculating the combination index (CI). Normalized isobolograms demonstrated a combination of *AC* and 5-FU synergistically suppressed the cell viability of colon cancer cells. CI values < 1 denotes synergy.

*AC* has been shown to have adjunctive effects of enhancing radiation therapy in oesophagal cancer patients [30]. Until now, the effect of *AC* on colon cancer cells has not been yet explored. For a better understanding of the effects of *AC* on patients with colon cancer, we applied Chou-Talalay’s combination index (CI) theorem for evaluation of combination effects [31] of *AC* and 5-FU. Furthermore, we examined the chances of *AC* synergies with 5-FU to suppress the colon cancer cells viability. Isobolograms were generated and assayed by applying different concentrations combination of *AC* and 5-FU, according to their IC50 values at which drugs were equipotent on respective cell lines. Data analyzed using CompuSyn an open-source software as per their explained methods. Several combinations of *AC* and 5-FU found to inhibit colon cancer (SW480, SW620 and HCT116) cells viability synergistically (CI index < 1, in bold, Figure 3). 

### 3.4. AC Treatment Effect and Colon Cancer Signaling Pathway 

**Figure 4:**
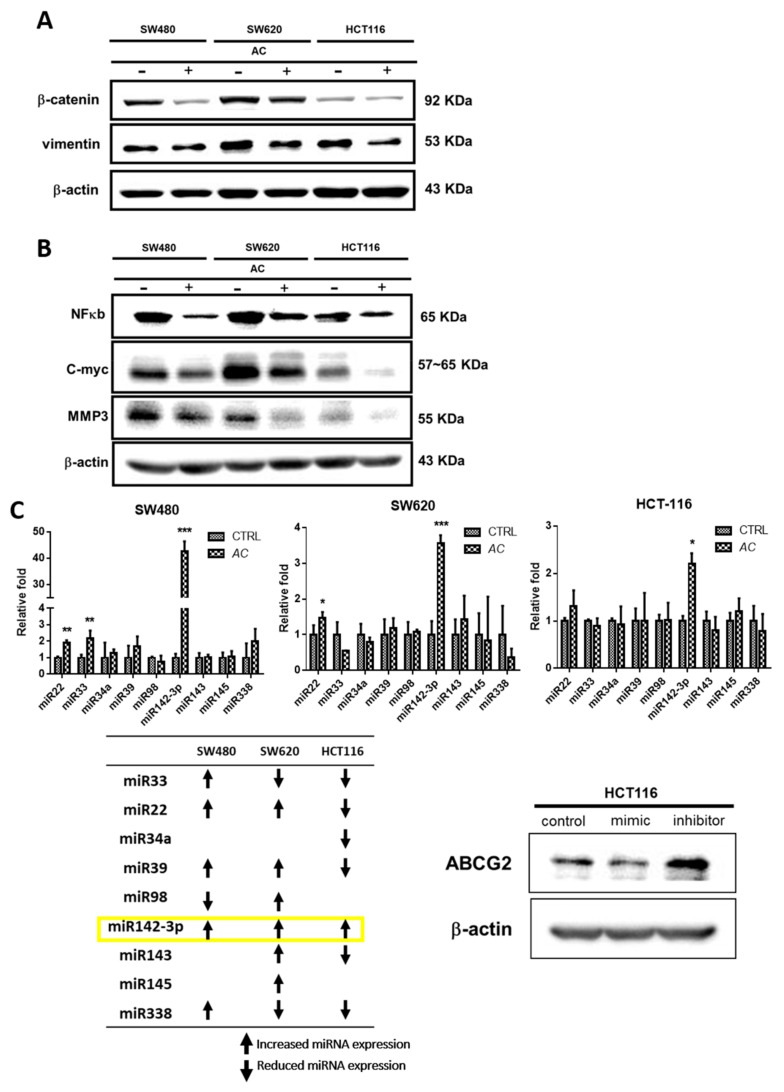
Impact of *AC* on the signaling pathway of CRC cell lines. *AC* (100 μg/mL) treatment were given to CRC cells for 24 h. (**A**) *AC* significantly decreases the EMT associated β-catenin and vimentin gene expression, (**B**) suppression of oncogenic markers NF-κb, C-myc and MMP3, and (**C**) upregulation of miRNA expression, specially miR142-3p, and miR142-3p expression negatively regulates ABCG2. * *p* < 0.05, ** *p* < 0.01 and *** *p* < 0.001.

Colorectal cancer connected with a higher recurrence rate and metastasis. Association between inflammatory gene and colorectal cancer has been shown through epidemiological data. A critical feature of these cancer cells has the ability to disintegrate the extracellular matrix (ECM) and basement membranes, largely mediated by matrix metalloproteinases (MMPs) [32]. Previous studies also have shown *AC* plays an important role in anti-inflammatory and anti-cancer in different cancers and animal models [19,33]. Our results also determine that *AC* mediated anti-cancer effects in CRC’s were associated with the decreased expression of EMT related β-catenin and vimentin genes (Figure 4A). Additionally, *AC* treatment also inhibited the expression of NF-κb and C-myc; and MMP3 across all three-colon cancer cell lines (Figure 4B). Furthermore, screening of miRNAs regulating gene expression after *AC* treatment was validated by q-PCR (Figure 4C). Increased miRNA expression, especially miR142-3p was expressed in colon cancers cells after *AC* treatment, and ABCG2 genes over-expression is importantly correlated with multidrug resistance mechanism associated with tumorigenic stem cells [34]. ABCG2 expression observed negatively regulated by miR142-3p (Figure 4C). Collectively, *AC* treatment on CRC cells induces the miR142-3p expression and it negatively regulates ABCG2.

### 3.5. AC treatment Inhibits Tumor-Initiating Capacity

**Figure 5:**
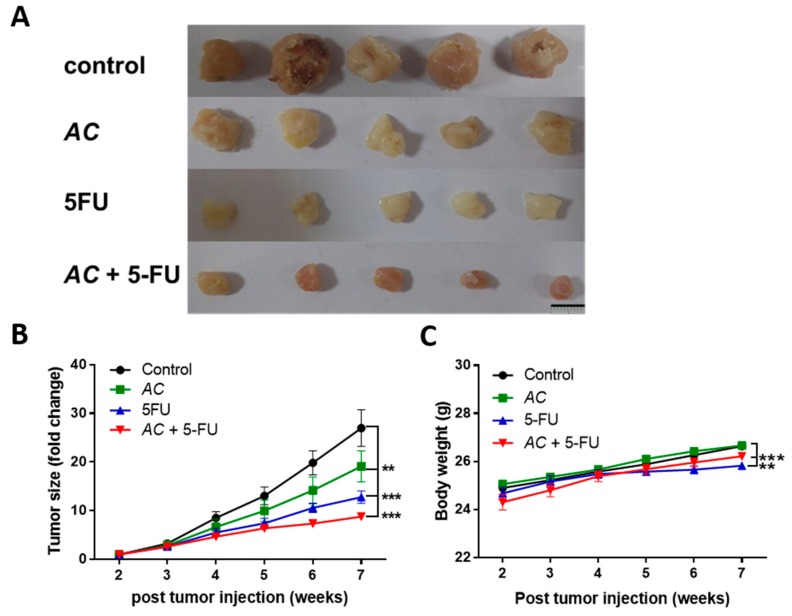
In-vivo tumor inhibitory effect of *AC*. (**A**) Photographs of human colon cancer cells, DLD-1 (1 × 10^6^ cells/injection, subcutaneous) were injected into NDO/SCD mice for establishing tumor xenograft model. When tumor size became palpable, mice were separated into four groups: Vehicle control, *AC* (50 mg/Kg), 5-FU (10 mg/kg), and combination *AC* + 5-FU (30 mg/kg 5-FU + 10 mg/kg *AC*). (**B**) Evaluation of tumor suppressive effect, synergistic effect of *AC* + 5-FU significantly decreases the size of tumor in the combination treatment group followed by *AC* and 5-FU individual treatment. (**C**) Averaged body weight of the animals, there is no negative impact of treatment on the body weight of animals, except for the 5-FU group. * *p* < 0.05, ** *p* < 0.01, and *** *p* < 0.001.

*AC* enhanced inhibition of tumor-initiating capability tested in vivo. Naïve DLD-1 colon cancer cells (1 × 10^6^ cells/injection) were subcutaneously injected into NOD/SCID mice to establish the xenograft models. After weeks of follow-up, mice were grouped into four different groups when tumor size became noticeable based on their treatment stratification. We found that combine treatment of *AC* + 5-FU group has got the best effect of tumor growth inhibition in comparison with other treatment arms. The size of tumor was measured for each treatment groups; a significantly lower tumor size was found in combines *AC* + 5-FU treatment group, reflecting the combination treatment strategy suppressed tumorigenesis (Figure 5A). This was also reflected in the changes in tumor weight across the treatment arms (Figure 5B). 

## 4. Discussion

To enhance the tumoricidal effect, but at the same time limiting the resistance to chemotherapy has long been approach and the primary task in cancer therapy. Numerous findings and studies have given the clue that some of the natural products holds the potential to achieve these objectives [35,36]. *AC* has been associated to inhibit liver cancer cells proliferation when used in combination with chemo-agent through suppression of Multi-Drug Resistance (MDR) genes expression and inhibition of COX-2 dependent pathway of phospho-AKT (p-AKT) [37]. *AC* treatment described to possess anti-cancer activities, but specific focus on its inhibitory effect on colon cancer stemness and drug resistance, and its modelling with a potential clinical application with 5-FU had seldom been reported. 

Escape of apoptosis is one of the key hallmarks of cancers that contribute to the cancer progression, as well as drug resistance in cancer [38]. Today’s researchers are more focused on finding natural products with apoptosis-facilitating potentials because that might have the likelihood in the development of medications for cancer and inflammatory diseases [39]. Preventing the creation of oxidative stress and cell death fund to be associated with high levels of *AC* containing flavonoids, terpenoids and polyphenolics [40]. Besides, *AC* regulation on the anti-apoptotic pathway was reported to include PI3K/Akt and activator of transcription (STAT), through the over-expressed EGFR in colorectal tumors [41]. 

Our study results show, *AC* significantly inhibits the growth and development of colon cancer cells in vitro (Figure 1A). Treatment with *AC* subsequently induces the apoptosis of colon cancer cells (SW480, SW620, and HCT 116) as demonstrated with Annexin-V assay (Figure 1C). The increased apoptosis was directly associated with increased expression of cleaved PARP (pro-apoptotic marker) (Figure 1D). Traditional cancer treatment is applying chemotherapy to inhibit and induce apoptosis of the hastily proliferating cancer cells [42]. However, cancers grown from a clone of heterogeneous malignant cell subpopulations with diverse characteristics and functions do escape our immunological surveillance, which resultants leads to cancer recurrence and metastasis after the primary response to chemotherapy [43,44]. CSC’s theory provides an alternative elucidation for the above treatment refractoriness of several cancers [45]. CSC’s are the populations of cancer cells with stem cell-like features are tumorigenic, self-renewing and more resistant to chemotherapeutic agents than other cancer cells [46,47]. Considering the emergent role of colon cancer stemness in chemotherapeutic resistance, these markers have become new therapeutic targets for colon cancer patients. The stemness of colon cancer stem cells were found to be associated with higher expression of CD133, ALDH, NANOG, OCT4 and SOX-2, and these markers are found connected to the drug resistance of cancer cells [48,49,50,51,52].

In this study, decreased the number and inhibited self-renewal capacity of colon tumor spheres were seen for *AC* treated group under serum-deprived condition (Figure 2A). These *AC* mediated anti-cancer effects were related to the decreased expression of cancer-associated stemness marker β-catenin, SOX-2, and Nanog (Figure 2B). Furthermore, *AC* also down-regulates the EMT-related gene such as vimentin and MMP3 (Figure 4A) with the upregulation of miRNA expression of miR142-3p across all the three colon cancer cells (Figure 4C), implicating the key regulatory function of miR142-3p in colon cancer stemness and metastasis. The combination therapy strategy where natural agents and chemotherapy agents are combined is a cornerstone of current cancer therapy has gained wide acceptance and popularity these days. For example, combination of *Curcumin* had shown to enhance the growth inhibition effect of FOLFOX in colon cancer cells [53]. Combination of *Nigella sativa* seed oil extract with novel octahydropyrazino[2,1-a:5,4-a′]diisoquinoline derivative (OM-90) represents stronger efficacy and enhances the anticancer effect in gastric cancer patients [54]. Resveratrol, a natural cell proliferation inhibitor, works in synergy with 5-FU thus decreasing cancer cell viability and inducing apoptosis [55]. Combination therapy allows targeting to the multiple pathways involved in cancer and applying many different mechanisms to reduce the development of drug resistance [56]. By combining two therapeutic agents into a cancer therapy may results in a synergistic effect, reduced (antagonistic), or identical (additive) effect in comparison to their individual treatment effects. How does the researcher’s make a prediction? It is very important for researchers to assess the interaction mathematically between compounds before actual in vivo or clinical use. Chou-Talay combination index (CI) was applied based on the equation such as *CI* = *a*/*A* + *b/B* [57]. It is interpreted as an additive interaction between two drugs when CI is equal to 1, synergism when CI < 1, and antagonism when CI > 1 [58]. Here, we found that the combined use of *AC* increased the 5-FU sensitivity in colon cancer cells. Normalized isobolograms demonstrated that the combination of *AC* and 5-FU synergistically suppress the colon cancer cell viability (Figure 3). Similarly, the result of in-vivo experiments in the mouse model also reflected the same outcome, that when a combination of *AC* with 5-FU used it produced the significant tumor inhibitory effect (Figure 5A–C).

## 5. Conclusions

The results show that *AC* is a powerful synergistic adjuvant, it down-regulates numerous cancer stemness associated genes and augments antitumor efficacy of 5-FU by triggering the expression of apoptosis-related genes in colon cancer cells, as well as suppressing inflammation and metastasis-related genes through miR142-3p, thus maximizing the therapeutic potential for patients with colon cancer.

## Supplementary Materials

The following are available online at https://www.mdpi.com/2218-273X/9/8/306/s1, Table S1: Primer sequences of microRNA.
