# Supplementary material for: Antrodia cinnamomea Enhances Chemo-Sensitivity of 5-FU and Suppresses Colon Tumorigenesis and Cancer Stemness via Up-Regulation of Tumor Suppressor miR-142-3p
Source: Biomolecules. 2019 Jul 25;9(8):306. doi: 10.3390/biom9080306 (PMC6723279; doi:10.3390/biom9080306)
Supplement: Supplementary file 1 [file biomolecules-09-00306-s001.pdf]

Table S1: Primer sequences of microRNA

miRNA	QIAGEN catalog number
hsa-miR-33	MS00003304
hsa-miR-22-3p	MS00003220
hsa-miR-34a-3p	MS00009534
hsa-miR-39-3p	MS00008666
hsa-miR-98	MS00003367
hsa-miR-142-3p	MS00031451
hsa-miR-143	MS00008687
hsa-miR-145-3p	MS00008708
hsa-miR-338	MS00003990
